# Current Trends in Acetins Production: Green versus Non-Green Synthesis

**DOI:** 10.3390/molecules27072255

**Published:** 2022-03-30

**Authors:** Bakht Zada, Moonhyuk Kwon, Seon-Won Kim

**Affiliations:** Division of Applied Life Science (BK21 Four), ABC-RLRC, PMBBRC, Gyeongsang National University, Jinju 52828, Korea; bzada@gnu.ac.kr

**Keywords:** glycerol, acetins, green, non-green, chemical synthesis, enzymes, microbial synthesis

## Abstract

To utilize excess glycerol produced from the biodiesel industry, researchers are developing innovative methods of transforming glycerol into value-added chemicals. One strategy adopted is the conversion of glycerol into acetins, which are esters of glycerol that have wide applications in cosmetics, pharmaceuticals, food and fuel additives, and plasticizers and serve as precursors for other chemical compounds. Acetins are synthesized either by traditional chemical methods or by biological processes. Although the chemical methods are efficient, productive, and commercialized, they are “non-green”, meaning that they are unsafe for the environment and consumers. On the other hand, the biological process is “green” in the sense that it protects both the environment and consumers. It is, however, less productive and requires further effort to achieve commercialization. Thus, both methodologies have benefits and drawbacks, and this study aims to present and discuss these. In addition, we briefly discuss general strategies for optimizing biological processes that could apply to acetins production on an industrial scale.

## 1. Introduction

All chemical compounds or substances used for commercial purposes are either natural (produced by plants, animals, and microbes) or synthetic (produced by chemists and engineers) [1]. The extraction of natural products from their native source is regarded as cleaner and more sustainable as compared to chemical synthesis, especially when green process concepts and principles are followed and utilized [2,3]. Advanced effective and selective approaches make it simple to extract or isolate a specific natural product from its natural host [2,3,4]. However, there is no such shortcut available for non-natural products, which need to be synthesized either by traditional chemical methods or by biological processes. Even a natural product may require synthesis because of its inefficient extraction from a natural source, prolonged extraction time, low-quality extraction, unit operations under harsh conditions, higher energy consumption, economical costs, or high quantity of waste generation. Additionally, chemical or biotechnological synthesis is often required to convert surplus industrial byproducts into value-added compounds such as the transformation of surplus glycerol generated during biodiesel production into value-added chemicals. Generally, products of interest are synthesized by traditional chemical methods because these methods have some advantages, such as scalability and economic feasibility. However, environmental pollution due to the employment of toxic solvents and chemical reagents and the release of hazardous byproducts are serious consequences of this approach.

**Figure 1:**
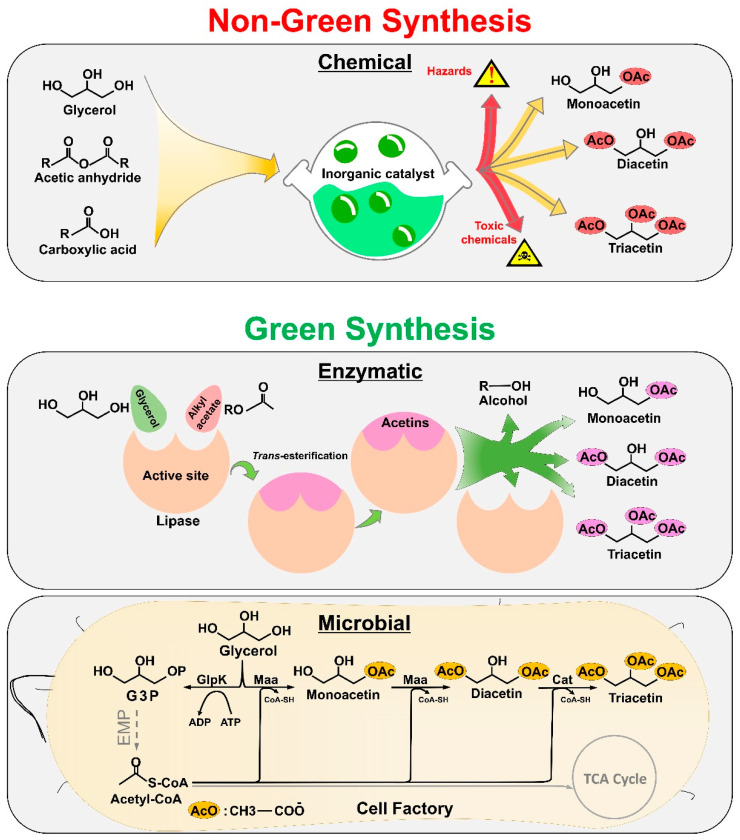
Acetins synthesis by “green” and “non-green” methods. The non-green synthesis of acetins involves traditional chemical methods that use inorganic catalysts (upper panel). The green synthesis of acetins involves enzymatic and microbial methods (middle and lower panel). The abbreviations are as follows: OAc, acetate group; Maa, maltose *O*-acetyltransferase; Cat, Chloramphenicol *O*-acetyltransferase.

Mono-, di-, and triacetin are non-natural compounds and therefore require synthesis [5,6,7,8,9]. Due to their great importance in a range of industrial applications, several methods have been described for their production. The synthetic methods are presented here as “green and non-green” (Figure 1). Both green and non-green methods have their benefits and limitations. The traditional chemical method, which is a non-green synthetic method of creating acetins, is based on the esterification of glycerol with carboxylic acid or acetic anhydride [10,11,12,13,14,15]. A high rate of conversion of glycerol to acetins is achieved, and the process is productive and has been commercialized. Nevertheless, there are several drawbacks associated with this approach. The conventional chemical routes for acetins synthesis suffer from the need for hazardous substances such as acetic anhydride and acetic acid, a high reaction temperature and pressure, and the release of toxic intermediates. These can cause environmental problems [16,17,18,19]. Moreover, the acetins generated by traditional chemical methods contain toxic contaminants such as reaction solvents and radicals, which limits their applications in the pharmaceutical, food, and cosmetic industries due to them being unsafe for consumers. Due to the aforementioned issues, researchers have instigated a search for an eco-friendly, renewable method of acetins production, often termed “green” or biological synthesis (Figure 1). When using a green route, acetins are produced either with enzymes [20,21] or microbial cells as biocatalysts [22]. Biocatalysis is a mature and widely used green technology for the eco-friendly production of valuable metabolites and commodity chemicals [23]. Lipases are one of the most common biocatalysts for acetins synthesis via the *trans*-esterification reaction of glycerol with alkyl acetate at a moderate temperature [20]. Alkyl acetate is used as an acetate donor for the *trans*-esterification reaction and the most commonly used acetate donor is methyl acetate, a well-known stable material for lipase activity [24]. An alcoholic byproduct, methanol, is produced during the lipase-catalyzed *trans*-esterification reaction of glycerol with methyl acetate, which is less harmful to the environment [20]. Using enzymes as biocatalysts for acetins production is a safe alternative to conventional chemical synthesis. The disadvantages of this method are that the enzymes are expensive, not easily available, unstable, and sometimes depend upon expensive cofactors (colipase) [25]. Moreover, the *trans*-esterification reaction catalyzed by commercial lipase is reversible and the co-substrate, alkyl acetate, incurs additional cost [26]. Therefore, this method cannot support the feasible and economical production of acetins and is unfavorable for commercialization without further efforts being made. The second route of acetins biosynthesis is microbial production, which could be a highly sustainable and environmentally favorable approach. Recently, we developed a biological method of acetins production by engineering *E. coli* as a cell factory [22]. A titer of more than 25 g/L was achieved in *E. coli* using glycerol as a sole substrate [22]. Further efforts are required to improve the existing host strain or find other robust strains for high yield and productivity. Fermentation process optimization will guarantee a higher yield of acetins and may even reach the commercialization scale.

## 2. Importance and Applications of Acetins

**Figure 2:**
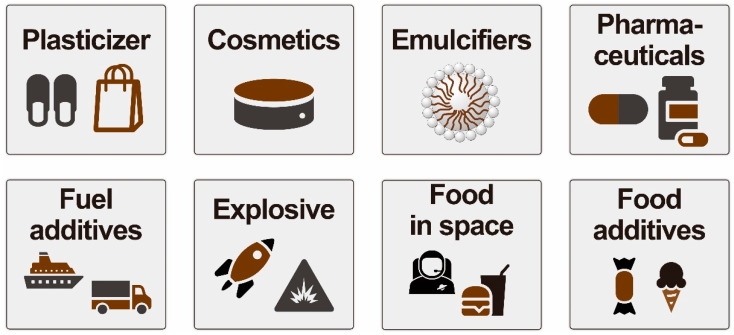
Common uses of acetins.

Approximately 10% of the crude glycerol produced globally is recovered from the biodiesel industry [15]. Due to the exponential growth in the production of biodiesel in recent years, glycerol has been oversupplied, and its production has surpassed two million metric tons, which could cause environmental issues. In addition, its value has decreased steadily. For the economic competitiveness of the biodiesel industry, increasing interest has been stimulated by research on the conversion of glycerol into value-added chemicals. One such strategy adopted is the transformation of glycerol into acetins. Acetins, also called glycerol acetates, exist in three forms: monoacetin (glycerol monoacetate), diacetin (glycerol diacetate), and triacetin (glycerol triacetate). The acetin form given is dependent on the number of the hydroxyl group of glycerol substituted with the acetyl group. Mono- and diacetin typically exist in two isomeric forms—1-monoacetin and 2-monoacetin—and, similarly, 1,2-diacetin and 1,3-diacetin. Acetins have garnered the most interest among the glycerol derivatives because of their extensive commercial applications (Figure 2). Their unique physiochemical properties such as stability, biodegradability, and water miscibility make them attractive for industrial applications. They are used as plasticizers, emulsifiers, stabilizers, solvents, space foods, cosmetics, pharmaceuticals, medicines, food additives, humectants, and vehicles for drug delivery systems [5,7,27]. They are also used as biofuel additives to improve viscosity and cold flow properties [8]. Monoacetin is used as a tanning agent in leather and for the production of explosives and smokeless powders [8]. Diacetin is used as a solvent for various dyes. Among the three products, triacetin is considered the most valuable and has been widely used in versatile applications. Therefore, the price of triacetin is comparatively high and stable, with demand growing by 5–10% yearly [28]. Triacetin is mostly used as a fuel additive due to its positive influences on fuel properties. A blending of 10% triacetin with biodiesel improves its viscosity and cold flow properties, enhances its octane rating, significantly improves engine performance, and reduces greenhouse gas emissions. Triacetin is also used as a solvent and as the acyl donor in isoamyl acetate synthesis [29,30], as well as in the synthesis of cinnamyl acetate from cinnamaldehyde [31]. The global demand for triacetin is more than 110,000 tons per annum [28]. It has been forecasted that the triacetin market will grow from 234.5 million USD to 309.9 million USD in 2022 and is expected to reach 362.1 million USD by 2026, with a CAGR (compound annual growth rate) of 4.8% during the forecast period [32,33]. Many chemical companies, such as Alfa Aesar; Lemon-Flex; Hefei TNJ Chemical Industry Co., Ltd.; Atanor S.C.A.; Spectrum Chemical Mfg. Corp.; Jiangsu Ruichen Chemical Co., Ltd.; Eastman Chemical Company; Lanxess AG; Yixing Kaixin Chemical Co., Ltd.; BASF SE; KLK OLEO; Polynt Group; Mosselman; and ReAct Chemical Co., Ltd., are involved around the world in manufacturing triacetin [14,34]. According to the “Triacetin Market Report 2021”, the key players in the triacetin market are North America (USA and Canada), Europe (UK, Germany, France, and the rest of Europe), Asia Pacific (China, Japan, India, and the rest of the Asia Pacific region), Latin America (Brazil, Mexico, and the rest of Latin America), and the Middle East and Africa (GCC and the rest of the Middle East and Africa) [35].

## 3. Non-Green Synthesis of Acetins

Numerous industrially relevant compounds are produced from glycerol via common synthetic approaches. In planning the chemical route for the conversion of glycerol into high value-added products, several factors are considered, such as the cost and availability of co-reactants, the amount of energy needed, and the cost of purifying the end products. Glycerol is converted into high-value products generally through esterification [34,36], etherification [37,38], oligomerization [39,40], hydrogenolysis [41,42], oxidation [43,44], acetalization [45,46], reforming [47,48], pyrolysis and gasification [49,50], dehydration [51,52], and carboxylation [53,54]. In recent years, researchers have paid great attention to the acetylation (esterification) of glycerol into acetins using various kinds of chemical catalysts. Based on catalytic acetylation reactions, which require acetate donors, several chemical methods have been described for acetins synthesis [7,15,55,56]. Most commonly, acetic acid or acetic anhydride is used as an acetate donor. In addition to catalysts and solvents, the synthesis of acetins by the acetylation reaction involves glycerol as a substrate and acetic acid or acetic anhydride as a co-substrate. The acetylation of glycerol to produce acetins proceeds either through homogeneous catalysts, which involve mostly mineral acids such as hydrofluoric acid, hydrochloric acid, sulfuric acid, nitric acid, phosphoric acid, *p*-toluene-sulfonic acid, pyridinium *p*-toluene-sulfonate, etc. [15,27,57,58,59,60], or heterogeneous solid acid catalysts such as ion-exchange and functionalized resins, activated carbon, functionalized biomass-derived carbon, metal oxides (mixed oxides and supported mixed oxides), silica (mesoporous or functionalized), zeolites and functionalized zeolites, heteropoly acids, and supported heteropoly acids [19,59,61,62,63,64,65,66,67,68,69,70,71,72,73]. A series of heterogeneous solid acid catalysts, including graphene oxide, Amberlyst-70, SnO_2_-based acid catalysts (MoO_3_/SnO_2_, SO_4_/SnO_2_), zirconia-based catalysts (WO_3_/TiO_2_-ZrO_2_, HSiW/ZrO_2_, HPW/ZrO_2_, and HPMoO_3_/TiO_2_-ZrO_2_), and silver-exchanged phosphortungstic acid (Ag_1_PW), have recently been reported for glycerol acetylation to acetins [74,75,76,77,78,79,80]. The product selectivity towards mono-, di-, or triacetin depends on the nature of the catalyst surface and the density and strength of the catalytic sites [81]. Like other chemical reactions, the acetylation of glycerol can be influenced by certain experimental parameters, such as reaction time, temperature, reactants, their molar ratios (acetic acid/acetic anhydride over glycerol), catalyst amount (load), surface acidity, and the stability of the catalyst [17].

The acetylation of glycerol with an acetate donor consists of three simultaneous consecutive reactions. The glycerol is first acetylated to monoacetin, involving one molecule of acetate donor; the monoacetin is converted into diacetin by the second acetylation with the donor, and subsequently, the diacetin is converted into triacetin by obtaining the final acetate group in the same way. The mechanism of glycerol acetylation depends upon the type of catalyst (Bronsted or Lewis acid) used for the reaction as well as the nature of the co-reactants, i.e., acetic acid or acetic anhydride. The reaction mechanism of acetylation in the case of acetic acid as a co-reactant is generally completed in three consecutive steps. First, protonation of the carbonyl group of the acetic acid occurs by a strong acid catalyst that generates a stable intermediate called acylium ion [17]. The resultant acylium ion is more susceptible to nucleophilic attack; therefore, in the second step the hydroxyl group of the glycerol, which acts as a nucleophile, attacks the acylium ion, producing a tetrahedral intermediate called hemiacetal, which affords two isomeric cyclic acetals by different pathways. Finally, the hemiacetal loses a water molecule, resulting in monoacetin [17]. Two different monoacetin isomers (1-monoacetin or 2-monoacetin) can be generated during this step. The monoacetin undergoes two consecutive reactions with acetic acid, producing di- and triacetin [77,82,83,84,85]. The acetylation of glycerol with acetic anhydride proceeds through two possible mechanisms [17,86,87,88]. In the first plausible mechanism, a carbonyl oxygen atom of acetic anhydride is protonated by a strong acid, thereby generating a positive center, which is attacked by a nucleophile (hydroxyl group of glycerol) to form a tetrahedral intermediate [17]. In the second plausible mechanism, the acidic site within the catalyst pores adsorbs acetic anhydride and forms a stabilized intermediate/acylium ion (acylation) along with the loss of an acetic acid molecule. Monoacetin is produced when the hydroxyl group of glycerol attacks the carbonyl group of the intermediate. Di- and triacetin are produced by repeating the same steps [17]. The last conversion reaction, i.e., triacetin production, is unsatisfactory [89] due to the comparatively lower standard Gibb’s free energy of the primary and secondary reactions (19.15 and 17.80 kJ/mol) compared to the tertiary reaction (55.58 kJ/mol) [90], and thus the yield of triacetin is lower than mono- and diacetin in the reaction mixture. Ionic liquid-based [91] and microwave-assisted acetylation of glycerol to acetins using activated natural zeolite have also been reported [64]. Due to more industrial applications and the demand for triacetin over mono and diacetins, several special methods have been described for its preparation at high yields [7,15]. These methods involve the acetylation of glycerol into monoacetin with acetic acid and the subsequent acetylation of monoacetin into di- and triacetin using acetic anhydride (instead of acetic acid) [15].

Both homogeneous and heterogeneous catalysts are extensively used for acetins synthesis on an industrial scale [7,15,55,56,57,59,62,92,93,94]. Homogeneous catalysts obtain a higher conversion rate and yield and are thus often preferred [16]. The esterification reaction of glycerol with acetic acid via homogeneous catalysts is less desirable because it requires harsh reaction conditions, exorbitant acetic acid consumption, a huge catalyst concentration, a higher reaction temperature, and a longer reaction time [16]. The electricity consumption in terms of reaction time demonstrated the highest environmental impact amongst other operating factors. The use of mineral acids as catalysts is usually limited by several major technical and environmental drawbacks, including product purity, reactor corrosion, and large waste amounts [95]. Therefore, due to these disadvantages, efforts have generally shifted to the use of heterogeneous catalysts that are considered less toxic, highly selective, easy to separate, relatively more sustainable, and favorable for the environment. The major advantage of heterogeneous catalysts is that it affords scientists the ability to manipulate the surface area and the acid density. In addition, heterogeneous catalysts are reusable, aiding their industrial applications for acetins synthesis. However, the active site leaching causes deactivation, instability, poor regeneration ability, and low turnover frequency. Moreover, undesirable reactions such as oxidation, dehydration, the *inter*-esterification occur [96]. Heterogeneous catalysts also suffer from high solubility in polar media and low specific surface areas.

The traditional chemical methods are highly efficient [97,98,99,100] in terms of their conversion rate, yield, and productivity [79,101,102], but suffer from environmental and technical drawbacks [103]. As mentioned above, the final products generated contain mostly mono- and diacetin (low-value product) with remarkably low triacetin (high-value product) levels. Both acetic acid and acetic anhydride are widely used as co-reactants in chemical synthesis methods for acetins, but there is no study comparing the economic and environmental sustainability of the acetic acid–glycerol and acetic anhydride–glycerol acetylation pathways. It is thus difficult to conclude which chemical route has better potential for acetins production on an industrial scale. The price of the co-reactants, acetic acid and acetic anhydride, are 160 and 170 USD per liter, respectively, if purchased from Sigma Aldrich. The use of these reactants becomes uneconomical for the mass production of acetins. Besides, the high explosion potential of acetic anhydride makes it unsuitable for manufacturing [28]. Therefore, a sustainable process is needed for acetins production to turn from conventional non-green synthesis into modern green synthesis to overcome the harmful consequences of a chemical process. The green synthesis of acetins could have less of a burden on the environment; thereby making it an economically and environmentally sustainable process.

## 4. Green Synthesis of Acetins

In modern science, “green synthesis” has gained extensive attention as a sustainable, reliable, and eco-friendly approach to synthesizing a wide range of platform chemicals and products. Green synthesis is regarded as an important tool to reduce the destructive effects associated with traditional chemical methods and thus to avoid the production of unwanted and harmful materials. Glycerol acts as a precursor for the production of a large number of commodity chemicals, but the synthesis of value-added compounds from glycerol by biological methods has recently been considered. As discussed above, the conventional conversion of glycerol to acetins is performed with common homogeneous or heterogeneous chemical catalysts. To make the production greener, the biological synthesis of acetins is of considerable importance over conventional approaches. Biological synthesis, which involves the use of enzymes and microorganisms, has been widely used for the production of various kinds of commercial products. The biological production of acetins has several advantages over the traditional chemical synthetic methods. For example, it provides high quality and safe products in an environmentally friendly way. Here, we briefly summarize the current advances in research on the green synthesis of acetins.

### 4.1. Enzymatic Synthesis of Acetins

Lipases have been applied for the synthesis of a wide variety of glycerol derivatives, including glycerol carbonate, etc. Acetins are prepared by the *trans*-esterification reactions (the exchange of groups between ester and alcohol) of glycerol or triglyceride catalyzed by lipases [20,104,105]. An acetate donor is needed for the *trans*-esterification reaction. The acetate donor should not affect the lipase stability and should be able to react at a moderate temperature. A variety of acetate donors are used for the *trans*-esterification reaction converting glycerol to acetins, including acetic acid, vinyl acetate, ethyl acetate, acetic anhydride, and methyl acetate [21]. Among different acetate donors, methyl acetate is a well-known stable material for lipase activity [106]. An alcoholic byproduct, methanol is generated during this reaction. The solvent and methyl acetate are reused after removing the target product (acetins) and the byproduct (methanol) from the reaction medium. Generally, oil-jacketed columns and molecular sieves are used for the separation of acetins and methanol, respectively [107,108]. Experimental findings have shown that lipases catalyze esterification reactions by the ping-pong bi-bi mechanism [109,110,111,112]. Thus, the enzymatic mechanism from methyl acetate and glycerol in the lipase-mediated acetins synthesis could be proposed as follows: Firstly, methyl acetate [MA] initially binds to lipase [LI] forming lipase-methyl acetate [LI-MA]. Secondly, the [LI-MA] subsequently isomerizes to an acetyl-lipase intermediate [LI*] by releasing methanol [MOH]. Thirdly, glycerol [Gly] binds to the [LI*] forming acetyl-lipase complex [LI*-Gly]. Fourthly, the [LI*-Gly] forms lipase-acetin complex [LI-AC]. Finally, the [LI-AC] produces acetins [AC] and free lipase [LI].

Lipases used for *trans*-esterification reactions are either classified according to their regioselectivity, such as nonspecific lipase, 1,3-selective lipase, and 1,3-specific lipase, or immobilization [112]. The immobilized lipases are more stable in organic solvents at high temperatures than non-mobilized lipases; however, the immobilized lipases are more expensive. The regioselectivity of lipase determines the *trans*-esterification reaction type [113,114]. Lipases such as Novozym 435, Novozym CALB L, Lipase AK, Lipase F-AP15, Lipase PS-DL, Lipozyme TL IM, and Lipozyme RM IM are potentially active for acetins synthesis. Novozym 435, a nonspecific lipase, and Lipozyme RM IM, a 1,3-selective lipase, have been used and conversion rates of 36.11 and 1.93%, respectively, were achieved [115]. By optimizing the reaction conditions for Novozym 435, conversion rates of 95.0% and 85.2% for pure and crude glycerol, respectively, were achieved [20]. Using *Triticum aestivum* lipase, a 65.93% glycerol conversion rate was achieved after 15 h [105]. Lipases from *Candida rugosa* OF; *Mucor javanicus*, LMJ, LOF; porcine pancreas, LPP; *Pseudomonas cepacea*, LPsC; *Pseudomonas sp.*, LPs; *Candida antarctica*, LCA; and *Candida cylindracea*, LCC were screened for acetins synthesis in their immobilized forms on acrylic resin [104]. The immobilized lipase from *Candida antarctica* resulted in the highest efficiency by producing a mixture of fatty acid esters and triacetin with a conversion rate of 80% [104]. The highest conversion rate was achieved with Novozym 435 from *Candida antarctica* immobilized on acrylic resin [21]. Unlike traditional chemical synthesis, there are several advantages to using lipases for acetins synthesis, such as lower energy requirements, less waste generation, higher quality and purer products, higher stability of catalysts (Novozym 435 can be reused more than 100 times), eco-friendliness, and no hazardous chemicals [104,116,117,118].

The lipase-based synthesis of acetins has a promising future. However, there are certain limitations to this approach, including a relatively high cost and a limited supply of lipases. Moreover, the final reaction mixture containing reactants, alcohol byproducts, and the target acetins increases the cost of the downstream process for the purification of acetins. Methyl acetate (co-substrate) incurs an extra cost in the lipase-based approach. Some lipases are sensitive to impurities, whereas crude glycerol contains various impurities such as salts, ashes, etc. Little work has been conducted on the development of robust lipase for acetins synthesis, and the production of acetins on an industrial scale is currently less effective. More novel lipases are still to be identified for the efficient production of acetins.

Another issue is the inhibition of lipase-catalyzed reactions by short-chain alcohols [119]. Since lipase has a considerably higher affinity toward short-chain alcohols than water, the alcohols molecules generated as a by-product in the acetins production could gradually replace water molecules on the lipase surface [119,120]. This could disrupt intra-protein hydrophobic interactions, resulting in the collapse of the enzyme structure following the irreversible deactivation of lipase. Moreover, alcohols have been regarded as reversible inhibitors by competitive binding to lipase. Thus, an improvement of lipase against short-chain alcohols inhibition is required by mining alcohol stable lipases, mutagenesis of existed enzymes, or process engineering. In addition to the kinetic and molecular inhibition, short-chain alcohol could also deactivate lipase by releasing it from solid support [119].

Immobilization of lipase using solid support could improve the enzyme stability, reuse, and recovery. Inorganic oxides such as silicon oxide, aluminum oxide, titanium oxide, and zirconium oxide have been widely applicated with high stability and sorption capacities [121]. However, the inorganic oxides have some limitations, such as lipase immobilization, including low affinity toward the enzymes; rigidity to a geometrical shape; and restricted biocompatibility resulting in lipase deactivation [122]. To improve lipase activity, hybrid and composite materials have been developed [109]. Magnetic particles, nanoparticles, mesoporous materials, ceramic materials, carbon nanotubes, and graphene are promising in the production of acetins using lipase [122,123].

### 4.2. Microbial Production of Acetins

Microorganisms grow in a wide variety of habitats and conditions and can utilize a broad range of substrates. Many bacteria, including *Escherichia*, *Klebsiella*, *Lactobacillus*, *Clostridium*, and others, efficiently metabolize glycerol [124,125]. In recent years, researchers have discovered a way to monetize extra crude glycerol from biodiesel manufacturers by converting it into value-added compounds through microbial fermentation [126,127]. Glycerol is extensively utilized for the microbial production of a variety of chemicals and products ranging from fuel additives to commodity chemicals, including 1,3-propanediol, 1,2-propanediol, docosahexaenoic acid, 1,3-dihydroxyacetone, citric acid, lactic acid, bio-ethanol, hydrogen, single cell oil, etc. [128,129]. The number of studies on the metabolic engineering of microbes for the preparation of chemicals and fuels from glycerol is increasing all the time, but the number of methods for the microbial engineering of glycerol into acetins is still limited. Several microorganisms, including *Klebsiella oxytoca*, *Enterobacter aerogenes*, and some *Enterobacter* species, have been reported to produce monoacetin in trace amounts [130]. The construction of novel pathways for target products is one of the most difficult tasks encountered by metabolic engineers. New enzymes must be developed for non-natural products such as acetins, which is a difficult task, but recent advances in synthetic biology and metabolic engineering are highly helpful. For instance, *E. coli* was recently metabolically engineered to produce acetins from glycerol as a substrate [22]. The acetins biosynthesis pathway was successfully constructed via the overexpression of enzymes, maltose-*O*-acetyltransferase (MAA), and chloramphenicol-*O*-acetyltransferase (CAT) (Figure 1). The titer of acetins in that study was stepwise increased from 0.04 g/L to more than 27 g/L using a variety of strategies, including heterologous gene expression, metabolic engineering, and culture optimization [22]. Acetins production via the microbial method does not require any co-substrate, while all other methods reported for acetins production are exclusively dependent on co-substrates (acetic acid, acetic anhydride, methyl acetate, etc.) as well as specific solvents for reactions. The successful construction of a microbial pathway for the green synthesis of acetins was reported for the first time; however, the conversion rate needs to be improved for commercial production. To achieve a successful transition from the laboratory-scale demonstration to the large-scale commercial production of acetins, three major performance parameters, the product yield (g/g of the substrate), the productivity (g/L/h), and the product titer (g/L), must be addressed. As acetins are one of the bulk chemicals, profit margins are razor-thin, so it is vital to optimize these three parameters to compete with traditional chemical synthesis. Here, we briefly explore the prospects for acetins production via microbial fermentation.

#### 4.2.1. Glycerol Utilization Engineering

The microbial host must be able to efficiently utilize the substrate to produce the desired product in high quantitates. The *E. coli* engineered to produce acetins utilized 4.1% out of 10% glycerol in the production medium [22]. It has been reported that glycerol is inefficiently utilized by *E. coli* and is known to trigger the carbon stress response, therefore rewiring glycerol metabolism in *E. coli* is highly successful in producing the high titer of the target metabolites [131,132]. Overexpression of genes involved in the glycerol utilization pathway aid in improving production. For example, the overexpression of the *glpK* gene improves the production of shikimic acid in *E. coli* from glycerol [133].

#### 4.2.2. Engineering Strain for Acetins Tolerance

Acetins seem to be inhibitory to the production host at high concentrations (our unpublished data). Industrial strains must be resistant to product accumulation in the production medium to achieve a high titer. The rational engineering strategy for improving the acetins tolerance is to express efflux pumps that could enhance the export of acetins. *E. coli* has been engineered by expressing efflux pumps for improving tolerance against inhibitory biofuels and carotenoids [134,135]. To boost acetins production, the same strategies might be helpful.

#### 4.2.3. Selection of Microbial Strain

One of the most critical components of the microbial production process is choosing the right host strain. To date, only *E. coli* has been engineered for acetins biosynthesis because it is a familiar model organism, relatively well studied, and easy to genetically manipulate. Several other robust microbial hosts that could be employed for the bioproduction of acetins include *Corynebacterium glutamicum*, *Bacillus* sp., *Clostridium* sp., *Pseudomonas* sp., and *S. cerevisiae*. A variety of methods have been implemented for genetic manipulations that optimize production. Recent developments in computational tools are very helpful for designing optimal and robust microbial strains that can produce acetins on a commercial scale.

## 5. Conclusions

**Figure 3:**
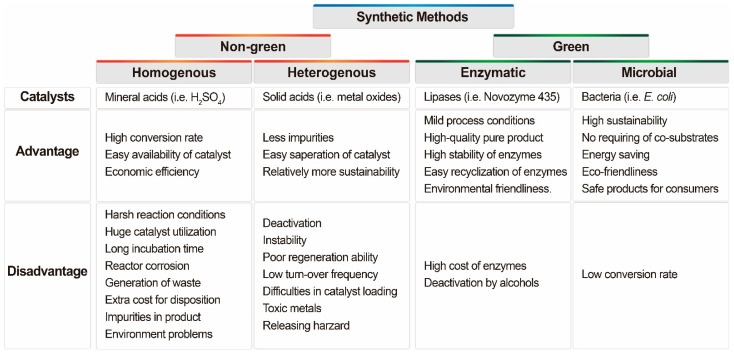
Advantages and disadvantages of different catalysts used in green and non-green synthesis methods of acetins.

Glycerol is transformed into acetins via two methods: the traditional chemical or the biological method. Glycerol is a byproduct of the biodiesel industry. It is preferable to convert it into value-added compounds in an environmentally friendly and sustainable manner rather than using a method that could harm the environment and be unsafe for consumers. When compared to non-green, traditional chemical synthesis methods, the green synthesis of acetins using biocatalysts or microbial fermentation is a very attractive proposition (Figure 3 and Table 1). The biosynthesis of acetins using crude glycerol from the biodiesel industry is mostly unknown, and consumer supply is largely reliant on chemical synthesis, which is hazardous to the environment and consumers. Further research into green synthesis is required to extend the current laboratory-based work to the industrial scale.

## Figures and Tables

**Table 1:** Glycerol conversion rates achieved to date using different synthetic routes for acetins production.

Synthetic Methods	Type of Catalysts	Catalysts	Conversion Rate (%)	Reaction Time (h)	Reference
Non-green	Mineral acid Ionic liquid Solid acid	Sulphuric acid [H-NMP] [HSO_4_] PrSO_3_H SAS	99.4 99.0 100	0.5 0.5 0.5	[91] [91] [71]
Green	Enzymes Microbes	Lipase *E. coli*	95.0 81.6 *	12 48	[20] [22]

* Both the glycerol backbone and acetate groups of acetins synthesis are derived only from glycerol (single substrate), whereas in other synthetic methods more than one substrate (i.e., acetic acid, acetic anhydride, or alkyl acetate in addition to glycerol) is required.

## Data Availability

Not applicable.
